# Effects and Mechanisms of Action of Acupuncture on Bone and Lipid Metabolism in a Rat Model of Postmenopausal Osteoporosis

**DOI:** 10.33549/physiolres.935700

**Published:** 2026-06-01

**Authors:** Sheng-Nan ZHANG, Chu-Chu ZHANG, Li-Hua JIANG, Gang OUYANG

**Affiliations:** 1 School of Acupuncture-Moxibustion and Tuina, Nanjing University of Chinese Medicine, Nanjing, Jiangsu, China; 2 Department of Traditional Medicine, Jiangsu Province Geriatric Hospital, Nanjing, Jiangsu, China

**Keywords:** Acupuncture, Postmenopausal osteoporosis, Bone metabolism, Lipid metabolism, Estradiol, Gut microbiota, cAMP-PKA-CREB pathway

## Abstract

This study investigated the effects of acupuncture on bone and lipid metabolism in a rat model of postmenopausal osteoporosis (PMOP). Thirty-two female Sprague-Dawley rats were randomly assigned to four groups: blank control, sham-operated, model (ovariectomy), and acupuncture treatment (ovariectomy plus acupuncture). Following treatment, bone mineral density (BMD), bone tissue morphological changes with quantitative histomorphometry, serum bone metabolism markers, estradiol (E2) levels at multiple time points, body mass index (BMI), blood lipid indicators, inflammatory factors, and gut microbiota composition were measured in each group. Acupuncture increased femoral BMD after adjusting for femoral shaft thickness (P<0.05) and improved trabecular bone structure, with significant rises in trabecular number, thickness, and bone volume fraction (P<0.05). Serum osteoprotegerin levels increased and osteoclast markers decreased. Serial E2 measurements showed progressive restoration from week 4. Acupuncture reduced BMI (P<0.05), with BMI changes negatively correlating with BMD improvements (r=−0.68, P<0.01). Lipid metabolism and inflammatory markers were significantly improved (P<0.05). Gut microbiota analysis indicated increased abundance of Lactobacillus and Bifidobacterium and decreased pathogenic bacteria (P<0.05). Liquid chromatography-mass spectrometry suggested acupuncture may promote osteogenic differentiation via upregulation of the cAMP-PKA-CREB pathway. Overall, acupuncture effectively enhances bone and lipid metabolism in PMOP rats through hormonal regulation, metabolic modulation, and gut microbiota optimization.

## Introduction

Postmenopausal osteoporosis (PMOP) is a common metabolic bone disease characterized by decreased bone mass and a deterioration of bone microstructure that lead to reduced bone strength and an increased risk of fractures [1]. With the global population ageing, PMOP has become a major public health concern, particularly affecting middle-aged and elderly women [2,3]. In China, the high prevalence of PMOP in women over 50 poses a substantial societal burden [4]. The primary cause of PMOP is estrogen deficiency, which disrupts bone metabolism by enhancing osteoclast activity and suppressing osteoblast function [5,6]. This hormonal imbalance is also associated with lipid metabolism disorders, weight gain and chronic inflammation, all of which contribute to osteoporosis progression [7,8].

Currently, treatment for PMOP mainly involves pharmacological and non-pharmacological therapies. Common drug treatments include bisphosphonates, selective estrogen receptor modulators, calcitonin and parathyroid hormone analogues [9]. In addition to conventional bisphosphonates, newer targeted drugs such as denosumab and romosozumab have also been widely used in clinical practice, showing significant efficacy in improving bone metabolism by inhibiting bone resorption or promoting bone formation. Although effective, long-term use may lead to side effects such as gastrointestinal discomfort, osteonecrosis and thrombosis [3,10]. Non-pharmacological treatments mainly include calcium and vitamin D supplementation, moderate exercise, smoking cessation and alcohol limitation [11]. However, these often fail to meet clinical needs, making it important to explore safe and effective alternative treatments, such as acupuncture.

As an important component of traditional Chinese medicine, acupuncture has shown good application prospects in the prevention and treatment of osteoporosis [4]. Acupuncture has the effects of regulating whole-body functions, promoting qi and blood circulation and balancing yin and yang, thus improving bone metabolism through multiple pathways [12]. Clinical studies have shown that acupuncture alone can alleviate pain symptoms in patients with osteoporosis [13]. Although previous studies have demonstrated that acupuncture can enhance bone density and improve bone microarchitecture in osteoporotic animal models, the precise underlying mechanisms remain unclear [14]. Specifically, the molecular pathways through which acupuncture concurrently regulates both bone and lipid metabolism, beyond the well-characterized Wnt/β-catenin signaling pathway, warrant further in-depth investigation.

In recent years, studies have found that abnormal lipid metabolism is closely related to the occurrence and development of osteoporosis [15]. The decrease in estrogen levels following menopause can lead to lipid metabolism disorders, which, in turn, affect bone metabolism [16]. Adipose tissue is not only an energy storage organ but also an important endocrine organ that can secrete various cytokines and hormones, including leptin and adiponectin, that can directly or indirectly affect bone metabolism [17]. In addition, lipid metabolism disorders can cause chronic inflammatory responses, and chronic inflammation is considered a key factor in the development of osteoporosis [18]. Furthermore, emerging evidence suggests that the gut microbiota plays a crucial role in bone metabolism through the gut-bone axis, with estrogen deficiency significantly altering gut microbial composition and function. Therefore, investigating the effects of acupuncture on bone metabolism, lipid metabolism, and gut microbiota in PMOP rat models is of great importance for elucidating the mechanisms of action of acupuncture in treating PMOP.

The cAMP-PKA-CREB pathway is an important cell signal transduction pathway that plays a key role in both bone metabolism and lipid metabolism [19,20]. The activation of this pathway can promote osteoblast differentiation and bone formation while inhibiting adipocyte differentiation [21,22]. However, it is not clear whether acupuncture regulates bone metabolism and lipid metabolism in PMOP through the cAMP-PKA-CREB pathway.

Based on the above background, this study establishes a PMOP rat model to observe the effects of acupuncture on bone density, bone tissue morphology with quantitative histomorphometry, bone metabolism markers, serial estradiol measurements, ovarian histology, body mass index with correlation to BMD, blood lipid indicators, inflammatory factors, and gut microbiota composition in rats. Metabolomic techniques are used to explore the potential mechanisms of action of acupuncture in treating PMOP, with a focus on changes in the cAMP-PKA-CREB pathway. This study aims to provide experimental evidence for using acupuncture to treat PMOP and offer new research directions for further elucidating its mechanisms of action.

## Materials and Methods

### Experimental animals

Thirty-two specific pathogen-free-grade female Sprague-Dawley (SD) rats, each weighing 250±20 g, were purchased from Wuhan Baisai Model Biological Technology Co., Ltd. (Wuhan, China; animal license number: SYXK [E] 2022-0124). The rats were kept in an environment with a temperature of 22–26 °C, a humidity of 50–60 % and a 12-h light-dark cycle, with free access to food and water. After 1 week of adaptive feeding, the experiment began. All operations complied with experimental animal ethics standards, and the experimental protocol was approved by the Experimental Animal Ethics Committee of Baisai Model Biological Centre (approval number: BSMS Animal [Fu] No. 2023-11-08A).

### Animal model establishment and grouping

#### Establishment of the postmenopausal osteoporosis rat model

A bilateral ovariectomy was used to establish a PMOP rat model, the specific steps of which were as follows. The anesthesia machine was initially set to input a 2 % isoflurane concentration. After 3 min, the concentration was gradually increased to 5 %. Once it was observed that the rat’s posture met the standard for completing the anesthesia induction, the concentration was adjusted to 2.5 % for maintenance. A modified longitudinal dorsal incision of 0.8–1 cm was made with the incision center at the intersection of a point one finger below the lower edge of the lowest rib and a point one and one-half fingers lateral to the femur. Subcutaneous tissue and muscles were separated, and the ovaries were located within the deep fat layer. The ovaries were ligated at the uterine horn and bilaterally removed. For the sham operation group, only part of the fat tissue around the ovaries was removed. For 3 days following surgery, the rats were administered 60,000 U of penicillin daily to prevent infection and fed 5 % glucose. From the 4^th^ day onward, feeding was conducted according to normal feeding standards. Vaginal smears were performed for 5 consecutive days after surgery, and the absence of estrous cycles indicated complete ovariectomy and successful castration.

#### Experimental grouping

Using a computer-generated random sequence, the 32 female SD rats were randomly assigned into four groups, with 8 rats in each group: (1) a blank control group (no treatment, regular feeding); (2) a sham operation group (removing only fat tissue around the ovaries to control for surgical effect, regular feeding); (3) a model group (bilateral ovariectomy, regular feeding); and (4) an acupuncture treatment group (bilateral ovariectomy followed by acupuncture treatment, regular feeding).

### Acupuncture treatment method

The acupuncture treatment group received acupuncture bilaterally at the following acupoints: Shenshu (BL-23), Zusanli (ST-36), Pishu (BL-20) and Sanyinjiao (SP-6). These points were selected based on previous studies reporting their efficacy in treating PMOP [14]. The acupoint location method referred to ‘Experimental Acupuncture’ [23]: (1) Shenshu: Located on both sides of the second lumbar vertebra, 6 mm lateral to the dorsal midline; (2) Zusanli: Posterior-lateral to the knee joint, approximately 3 mm below the head of the fibula; (3) Pishu: Between the ribs on both sides of the twelfth thoracic vertebra, 6 mm lateral to the dorsal midline; (4) Sanyinjiao: Directly superior to the tip of the medial malleolus of the hind limb by 10 mm. [23]. The rats were fixed in a custom-made acupuncture rat cage, and specially made rat acupuncture needles (HQA-004, Huanqiu) with a diameter of 0.25 mm and a length of 13 mm were quickly and accurately inserted to a depth of around 3–5 mm. After obtaining the needle sensation (a slight muscle twitching or limb retraction), the needles were retained for 20 min (with twirling every 5 min). Based on existing acupuncture PMOP animal experiments [14] and clinical acupuncture treatment experience, acupuncture was performed 5 days a week, once a day, for a course of 3 months.

### Specimen collection

Baseline measurements of body weight, body length, and serum estradiol (E2) levels were obtained from all rats before surgery. After 3 months of treatment, all rats were subjected to fasting for 12 h with free access to water and anesthetized. The anesthesia machine was initially set to input a 2 % isoflurane concentration. After 3 min, the concentration was gradually increased to 5 %. Once it was observed that the rat’s posture met the standard for completing the anesthesia induction, the concentration was adjusted to 2.5 % for maintenance. After weighing, 10 ml of blood was collected from the abdominal aorta of each rat, centrifuged at 3,000 rpm for 10 min to separate the serum and aliquoted and stored in a −80 °C freezer for testing. Following euthanisation, the femurs of each rat were quickly separated, and the surrounding soft tissues were removed. The right femurs were used for bone density and histomorphological examinations, and the left femurs were used for Western blot detection. Fresh fecal samples were collected from the colon immediately after euthanisation and stored at −80 °C for gut microbiota analysis.

### Detection indicators and methods

#### Bone mineral density measurement

A dual-energy X-ray bone densitometer (Lunar Prodigy, GE Healthcare, USA) was used to measure the bone mineral density (BMD) of the right femur of each rat at baseline (before surgery) and at the end of treatment (3 months). The femur was placed in 1-cm-deep water to simulate soft tissue. The scanning range spanned from the femoral head to the distal end of the femur, with a resolution of 1.0×1.0 mm. Each sample was scanned twice, and the average value was taken. To account for variations in bone size, femoral shaft thickness (cortical thickness at mid-diaphysis) was measured using a digital caliper (accurate to 0.01 mm), and BMD values were adjusted using the formula: Adjusted BMD = Raw BMD × (mean femoral shaft thickness/individual femoral shaft thickness).

#### Bone tissue morphology observation and quantitative histomorphometry

Each right femur was stained with hematoxylin-eosin (H&E), and the specific steps of this process were as follows: the femur was fixed in 4 % paraformaldehyde for 7 days, decalcified in 10 % EDTA for 4 weeks, dehydrated in gradient ethanol, cleared in xylene, embedded in paraffin and cut into 4-μm-thick continuous sections. After routine H&E staining, changes in trabecular bone structure were observed under an optical microscope.

For quantitative histomorphometric analysis, digital images of H&E-stained sections were captured at 200× magnification using a light microscope equipped with a digital camera (Olympus BX51, Japan). Three non-overlapping fields of view from the metaphyseal region of each femur were analyzed using Image-Pro Plus 6.0 software (Media Cybernetics, USA). The following parameters were measured according to standardized guidelines [24]: trabecular number (Tb.N, per mm), trabecular thickness (Tb.Th, μm), trabecular separation (Tb.Sp, μm), and bone volume fraction (BV/TV, %). All measurements were performed by two independent observers blinded to the experimental groups, and the average values were used for statistical analysis.

#### Detection of serum bone metabolism markers

Enzyme-linked immunosorbent assay (ELISA) was used to detect the levels of osteoprotegerin (OPG), receptor activator of nuclear factor-κB ligand (RANKL), β-collagen degradation products (β-CTX) and N-terminal propeptide of type I procollagen (PINP) within the serum. Commercial ELISA kits (R&D Systems, USA) were used with strict adherence to the instructions. Each sample was measured in triplicate, and average values were taken.

#### Serial estradiol measurements

Serum estradiol (E2) levels were measured at multiple time points: baseline (before surgery), and at weeks 2, 4, 6, 8, 10, and 12 of treatment. Blood samples (0.5 ml) were collected from the tail vein at each time point (except baseline and endpoint, which were collected from the abdominal aorta). E2 levels were determined using a highly sensitive ELISA kit (Elabscience, China; detection range: 3.13–200 pg/ml, sensitivity: 1.17 pg/ml, 1pg/ml≈3.67pmol/l) following the manufacturer’s instructions. All samples were measured in duplicate, and the average values were recorded.

#### Body weight and body mass index measurements

The weight and body length (length from nose tip to anus) of the rats were measured weekly. Body weight was measured using an electronic balance (accurate to 0.1 g), body length was measured using a vernier calliper (accurate to 0.1 cm) and BMI was calculated as follows: weight/length^2^ (g/cm^2^).

#### Detection of blood lipids and inflammatory factors

An automatic biochemical analyser (Hitachi 7600, Japan) was used to measure the serum levels of total cholesterol (TC), triglycerides (TG), low-density lipoprotein cholesterol (LDL-C) and high-density lipoprotein cholesterol (HDL-C). The ELISA method was used to detect the serum levels of interleukin-6 (IL-6) and tumour necrosis factor-α (TNF-α) using commercial ELISA kits (Abcam, UK) with strict adherence to the instructions.

#### Gut microbiota analysis

Bacterial genomic DNA was extracted from fecal samples using the QIAamp DNA Stool Mini Kit (Qiagen, Germany) according to the manufacturer’s protocol. The V3–V4 hypervariable regions of the 16S rRNA gene were amplified using universal primers (341F: CCTACGGGNGGCWGCAG; 805R: GACTACHVGGGTATCTAATCC). PCR products were purified and sequenced on an Illumina MiSeq platform (Illumina, USA). Raw sequencing data were processed using QIIME2 (version 2020.11). Quality filtering, denoising, and chimera removal were performed using DADA2. Amplicon sequence variants (ASVs) were classified taxonomically using the SILVA database (version 138). Alpha diversity indices (Shannon, Simpson, Chao1) and beta diversity (weighted UniFrac distance) were calculated. Linear discriminant analysis effect size (LEfSe) was used to identify differentially abundant taxa between groups (LDA score >2.0, P<0.05).

#### Metabolomics analysis

Liquid chromatography mass spectrometry technology was used to identify and quantitatively analyse serum lipid metabolites. Chromatographic separation was performed using an ACQUITY UPLC HSS T3 chromatographic column (Waters, USA), with a mobile phase A of 0.1 % formic acid aqueous solution and a phase B of 0.1 % formic acid acetonitrile solution. Mass spectrometry analysis was performed using a Q Exactive mass spectrometer (Thermo Fisher Scientific, USA), featuring an electrospray ionization source and alternating positive and negative ion mode scanning.

Data was processed using Compound Discoverer 3.1 software (Thermo Fisher Scientific, USA) for peak identification, peak alignment and peak area integration, and SIMCA-P 14.1 software (Umetrics, Sweden) was used for principal component analysis, partial least squares discriminant analysis (PLS-DA) and orthogonal PLS-DA. MetaboAnalyst 4.0 software was used for metabolic pathway analysis.

#### Western blot detection

Proteins were extracted from left femur tissue, and protein concentrations were determined using the bicinchoninic acid method. Equal amounts of protein (30 μg) were separated by 10 % sodium dodecyl sulphate polyacrylamide gel electrophoresis and transferred to polyvinylidene fluoride membranes (Millipore, USA). After blocking with 5 % skim milk for 2 h, the membranes were incubated overnight at 4 °C with the following corresponding primary antibodies: anti-p-PKA anti-p-PKA (Cat# ab32390, Abcam), anti-p-CREB (Cat# 81871-1-RR, Proteintech), antiPKA (Cat# PA5-106474, Thermo), anti-CREB (Cat#12208-1-AP, Proteintech) and anti-β-actin (Cat# ab8227, Abcam). After washing with tris-buffered saline with 0.1 % Tween® 20 detergent, the membranes were incubated with horseradish peroxidase-labelled secondary antibodies (Cat# 711-035-152, Jackson ImmunoResearch) at room temperature for 1 h. An enhanced chemiluminescence kit (Thermo Fisher Scientific, USA) was used for development, and images were captured using a ChemiDoc XRS+ system (Bio-Rad, USA). Beta-actin was used as an internal reference protein, and Image J software was used to analyze the grey values of the bands.

### Statistical analysis

Statistical analysis was performed using SPSS v. 27.0 software (IBM, Armonk, NY, USA). Measurement data were expressed as mean ± standard deviation (χ̄ ± s). Comparisons among multiple groups were performed using one-way analysis of variance, and pairwise comparisons were performed using a least significant difference *t*-test. Correlation analysis between BMI changes and BMD improvements was performed using Pearson correlation coefficient. Serial E2 measurements were analyzed using repeated measures ANOVA. Results were considered statistically significant at P<0.05.

### Institutional clearance

This study was conducted in accordance with the declaration of Helsinki. This study was conducted with approval from the Ethics Committee of Baisai Model Biological Center, approval number: BSMS Animal (Fu) No. 2023-11-08A.

## Results

### Effects of acupuncture on bone density and bone tissue morphology

**Fig. 1:**
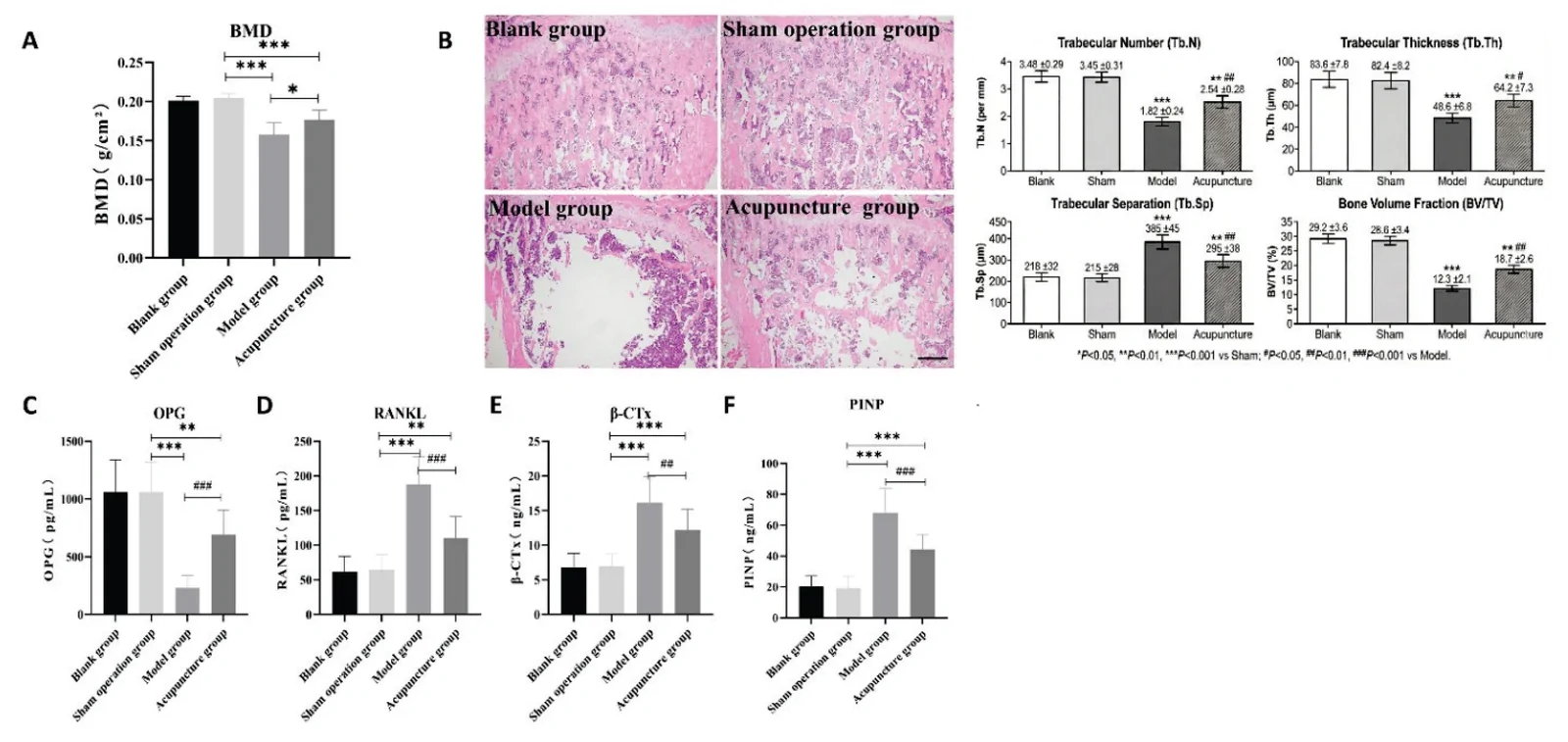
Effects of acupuncture on bone density, bone tissue morphology with quantitative histomorphometry, and bone metabolism markers in PMOP rats. (**A**) Comparison of femoral BMD adjusted for shaft thickness. (**B**) HE staining and quantitative histomorphometric analysis showing Tb.N, Tb.Th, Tb.Sp, and BV/TV. (**C–F**) Serum levels of OPG, RANKL, β-CTX, and PINP. Data are mean ± SD, n=8. * P<0.05, ** P<0.01, *** P<0.001; ^#^ P<0.05, ^##^ P<0.01, ^###^ P<0.001.

Femoral shaft thickness and bone metabolism markers were assessed in each group at baseline and at 3 months. No significant differences in baseline femoral shaft thickness were observed among the groups (P>0.05). At 3 months, model group rats showed decreased femoral shaft thickness compared to sham group (P<0.01), while acupuncture treatment partially prevented this reduction (P<0.05 vs. model group). At baseline, no significant differences in BMD were observed among groups (P>0.05). After 3 months, compared with the sham operation group, the raw femoral BMD of rats in the model group was significantly decreased (0.153±0.015 vs. 0.204±0.006, P<0.001). After adjusting for femoral shaft thickness differences, the acupuncture treatment group displayed significantly increased adjusted femoral BMD values (0.184±0.011 vs. 0.156±0.014, P<0.05) compared with the model group (Fig. 1A).

The H&E staining results showed that trabecular bone structure in the acupuncture treatment group was greatly improved compared with that of the model group (Figure 1B). Quantitative histomorphometric analysis revealed significant differences in trabecular bone parameters: Compared with the sham operation group, the model group showed significantly decreased Tb.N (1.82±0.24 vs. 3.45±0.31 per mm), Tb.Th (48.6±6.8 vs. 82.4±8.2 μm), and BV/TV (12.3±2.1 vs. 28.6±3.4 %), along with increased Tb.Sp (385±45 vs. 215±28 μm) (all P<0.001). Acupuncture treatment significantly improved these parameters compared to the model group: Tb.N increased to 2.54±0.28 per mm (P<0.01), Tb.Th to 64.2±7.3 μm (P<0.05), BV/TV to 18.7±2.6 % (P<0.01), and Tb.Sp decreased to 295±38 μm (P<0.01) (Fig. 1B quantitative panels).

### Effects of acupuncture on serum bone metabolism markers

Table S1 also presents the detailed statistical values of serum bone metabolism markers in each group of rats. Compared with the sham operation group, the serum levels of OPG in the model group were significantly decreased (P<0.001), and the serum levels of RANKL, β-CTX and PINP in the model group were all significantly increased (P<0.001). Compared with the model group, the serum OPG level in the acupuncture treatment group was significantly increased (P<0.001), whereas the RANKL, β-CTX and PINP levels were significantly decreased (P<0.01) (Fig. 1C–F).

### Serial estradiol measurements

**Fig. 2:**
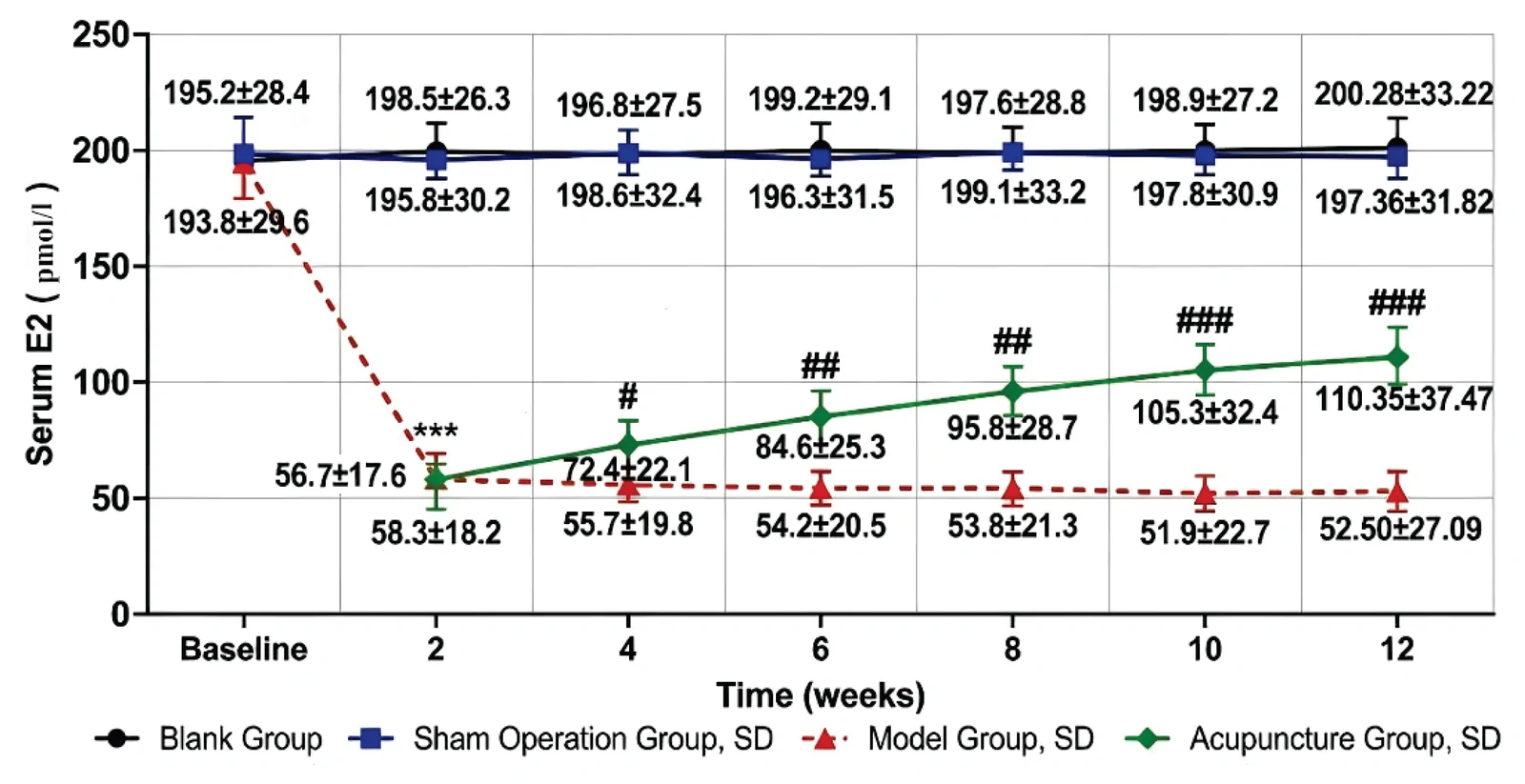
Serial estradiol E2 (pmol/l) measurements in PMOP rats. E2 levels were measured at baseline and weeks 2, 4, 6, 8, 10, and 12 during the treatment period. Data are mean ± SD, n=8. *** P<0.001 vs. Sham; ^#^ P<0.05, ^##^ P<0.01, ^###^ P<0.001 vs. Model group at corresponding time points.

Serial E2 measurements demonstrated distinct temporal patterns (Fig. 2): At baseline, all groups showed similar E2 levels (P>0.05). Following ovariectomy, E2 levels in both model and acupuncture groups dropped sharply by week 2, confirming successful estrogen depletion. From week 4 onwards, the acupuncture group showed gradual E2 elevation (week 12: 110.35±37.47 pmol/l), while the model group remained suppressed (week 12: 52.50±27.09 pmol/l, P<0.001). The blank and sham groups maintained stable E2 levels throughout the study (week 12: 200.28±33.22 and 197.36±31.82 pmol/l, respectively).

### Effects of acupuncture on BMI and correlation with BMD

**Fig. 3:**
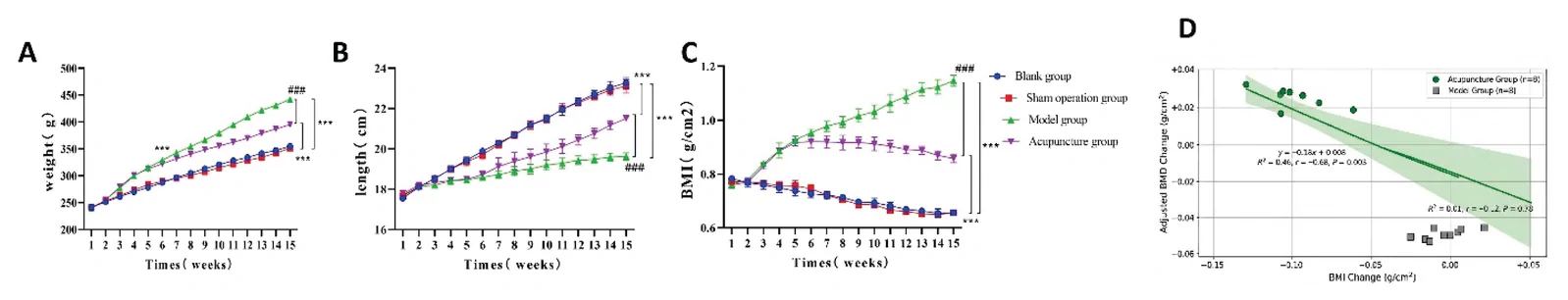
Effects of acupuncture on body weight, BMI, and correlation with BMD in PMOP rats. (**A–C**) Changes in body weight, body length, and BMI over time. (**D**) Correlation between BMI changes and adjusted BMD improvements. Data are mean ± SD, n=8. *** P<0.001; ^###^ P<0.001.

From 6 weeks after acupuncture treatment until the end of treatment, acupuncture considerably alleviated the body weight increase and the body length reduction in rats, resulting in a significant decrease in BMI (P<0.05) (Fig. 3A–C, Table S2).

Correlation analysis revealed a significant negative association between BMI changes and BMD improvements in the acupuncture group (Pearson’s r=−0.68, P=0.003, 95 % CI: −0.89 to −0.28). Specifically, rats showing greater BMI reduction exhibited larger increases in adjusted femoral BMD (R^2^=0.46). This relationship was not observed in the model group (r=−0.12, P=0.78) (Fig. 3D), suggesting that acupuncture-mediated metabolic improvements contribute to bone health enhancement.

### Effects of acupuncture on blood lipid levels and inflammatory factors

**Fig. 4:**
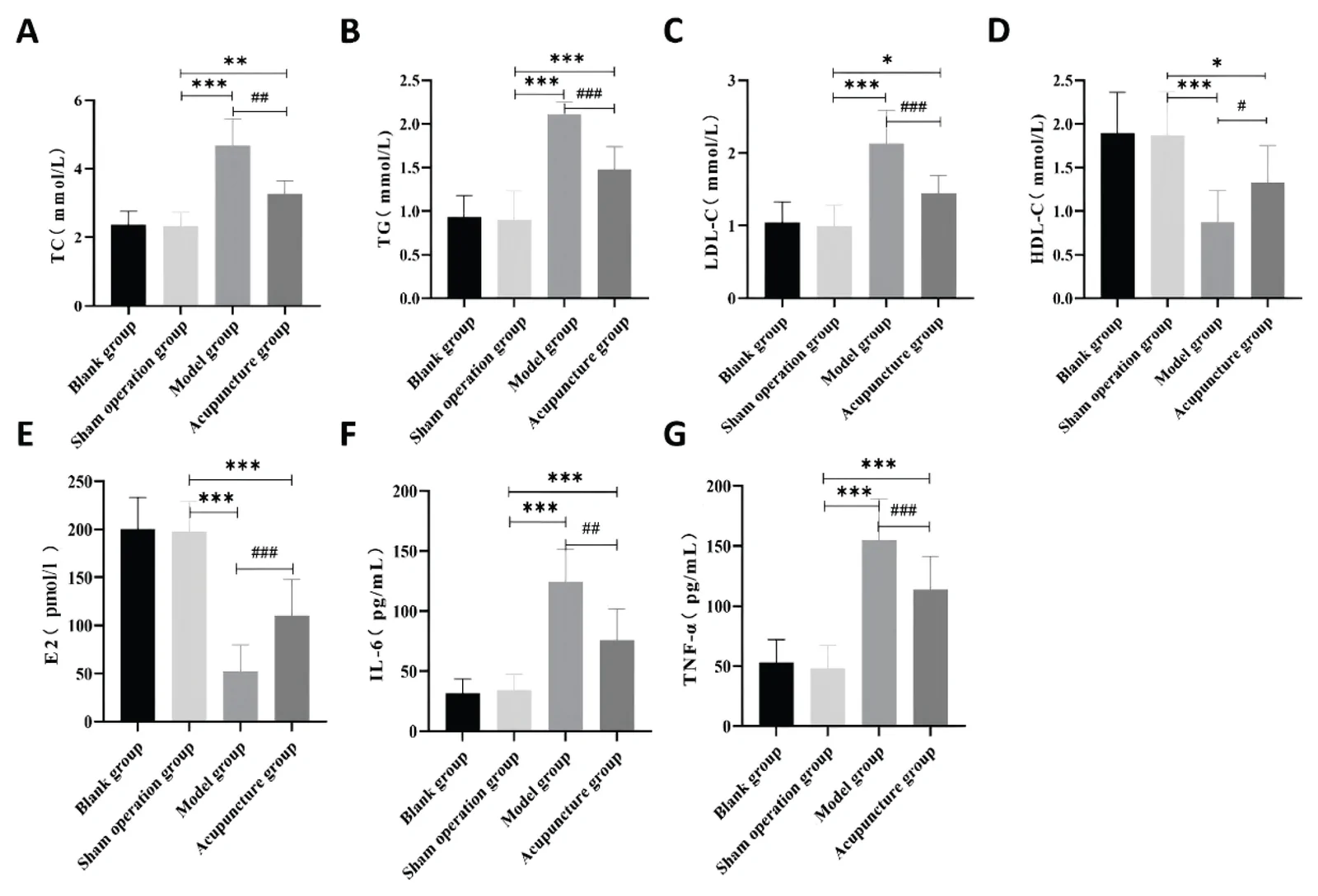
E2 (pmol/l) effects of acupuncture on blood lipid levels and inflammatory factors in PMOP rats. (**A–D**) Serum TC, TG, LDL-C, and HDL-C levels. (**E–G**) Serum E2, IL-6 and TNF-α levels. Data are mean ± SD, n=8. * P<0.05, ** P<0.01, *** P<0.001; ^#^ P<0.05, ^##^ P<0.01, ^###^ P<0.001.

Compared with the model group, acupuncture treatment significantly decreased serum TC (3.27±0.38 vs. 4.68±0.78 mmol/l, P<0.01), TG (1.48±0.26 vs. 2.11±0.14 mmol/l, P<0.001) and LDL-C (1.45±0.24 vs. 2.13±0.46 mmol/l, P<0.001) levels while elevating HDL-C (1.33±0.42 vs. 0.87±0.36 mmol/l, P<0.05) levels (Fig. 4A–D, Table S3). Acupuncture treatment also significantly decreased the inflammatory factors IL-6 (75.69±26.20 vs. 124.20±27.14 pg/ml, P<0.01) and TNF-α (113.54±27.80 vs. 154.85±34.28 pg/ml, P<0.001) levels (Fig. 4E–G, Table S3).

### Effects of acupuncture on gut microbiota composition

**Fig. 5:**
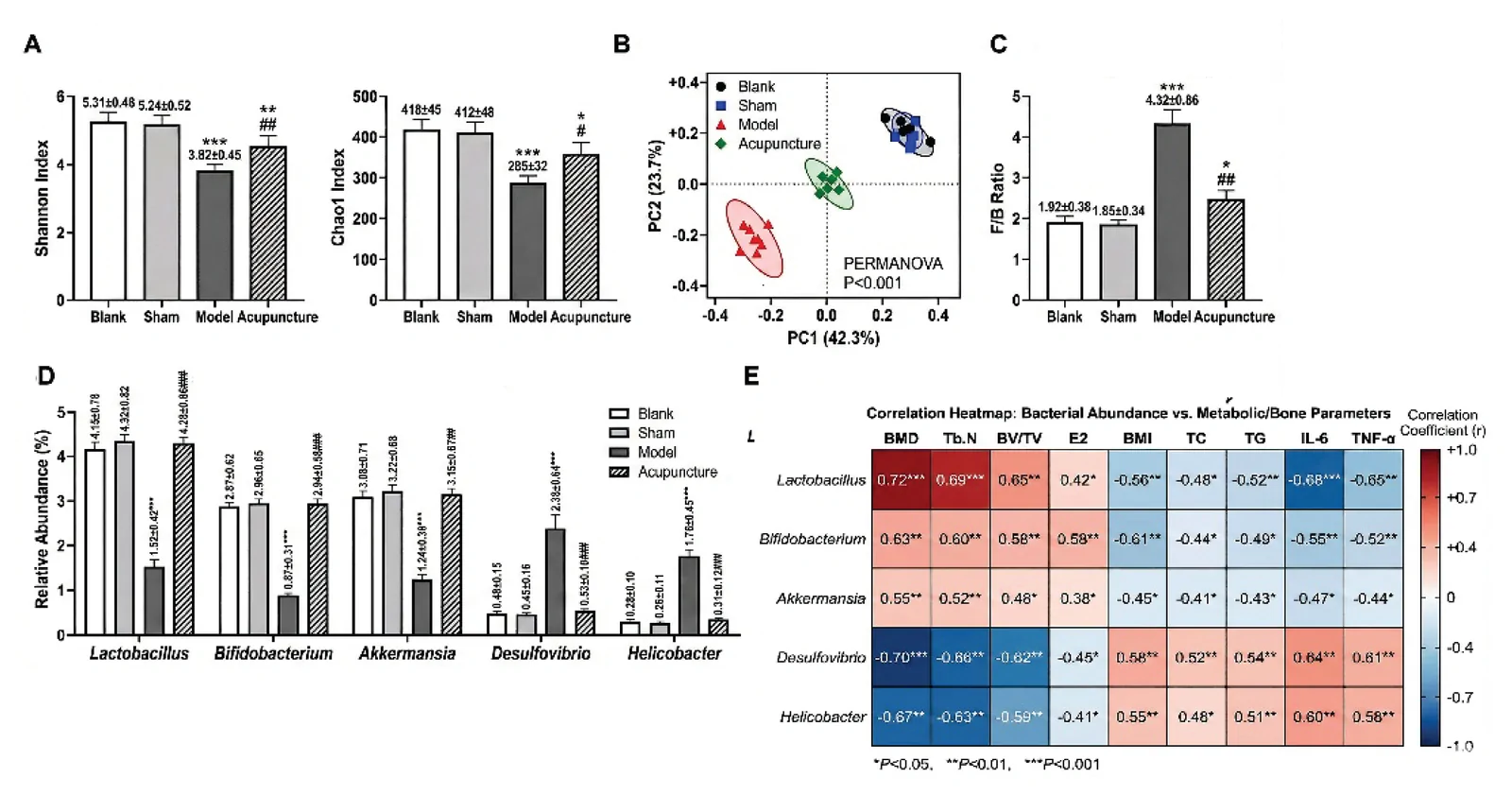
Effects of acupuncture on gut microbiota composition. (**A**) Alpha diversity indices (Shannon, Chao1). (**B**) PCoA plot of beta diversity. (**C**) Firmicutes/Bacteroidetes ratio. (**D**) Relative abundance of key bacterial taxa. (**E**) Correlation heatmap between bacterial abundance and metabolic parameters. Data are mean ± SD, n=8. * P<0.05, ** P<0.01, *** P<0.001 vs. Sham; ^#^ P<0.05, ^##^ P<0.01, ^###^ P<0.001 vs. Model group.

16S rRNA gene sequencing analysis revealed significant alterations in gut microbiota composition across experimental groups (Fig. 5). Alpha diversity analysis showed that the model group had significantly reduced bacterial diversity compared to the sham group (Shannon: 3.82±0.45 vs. 5.24±0.52, P<0.001; Chao1: 285±32 vs. 412±48, P<0.001). Acupuncture treatment partially restored alpha diversity (Shannon: 4.56±0.48, P<0.01 vs. model; Chao1: 358±41, P<0.05 vs. model) (Fig. 5A).

Beta diversity analysis using weighted UniFrac distances revealed distinct clustering of samples, with the acupuncture group showing an intermediate position between the sham and model groups (Fig. 5B). The Firmicutes/Bacteroidetes ratio, an important indicator of gut microbiota balance, was significantly elevated in the model group (4.32±0.86) compared to the sham group (1.85±0.34, P<0.001), and acupuncture treatment significantly normalized this ratio (2.48±0.52, P<0.01 vs. model) (Fig. 5C).

Compared to the model group, acupuncture significantly increased the relative abundance of beneficial bacteria including Lactobacillus (4.28±0.86 % vs. 1.52±0.42 %, P<0.001), Bifidobacterium (2.94±0.58 % vs. 0.87±0.31 %, P<0.001), and Akkermansia (3.15±0.67 % vs. 1.24±0.38 %, P<0.01). Conversely, acupuncture decreased potentially pathogenic bacteria such as Desulfovibrio (0.52±0.18 % vs. 2.38±0.64 %, P<0.001) and Helicobacter (0.31±0.12 % vs. 1.76±0.45 %, P<0.001) (Fig. 5D).

Correlation analysis revealed that Lactobacillus abundance was positively correlated with BMD (r=0.72, P<0.001), Tb.N (r=0.69, P<0.001), and BV/TV (r=0.65, P<0.01), and negatively correlated with IL-6 (r=−0.68, P<0.001) and TNF-α (r=−0.65, P<0.01). Bifidobacterium abundance showed positive correlation with serum E2 levels (r=0.58, P<0.01) and negative correlation with BMI (r=−0.61, P<0.01) (Fig. 5E). These correlations suggest that gut microbiota modulation may be an important mechanism underlying acupuncture’s therapeutic effects on bone and metabolic health.

### Effects of acupuncture on the cAMP-PKA-CREB pathway

**Fig. 6:**
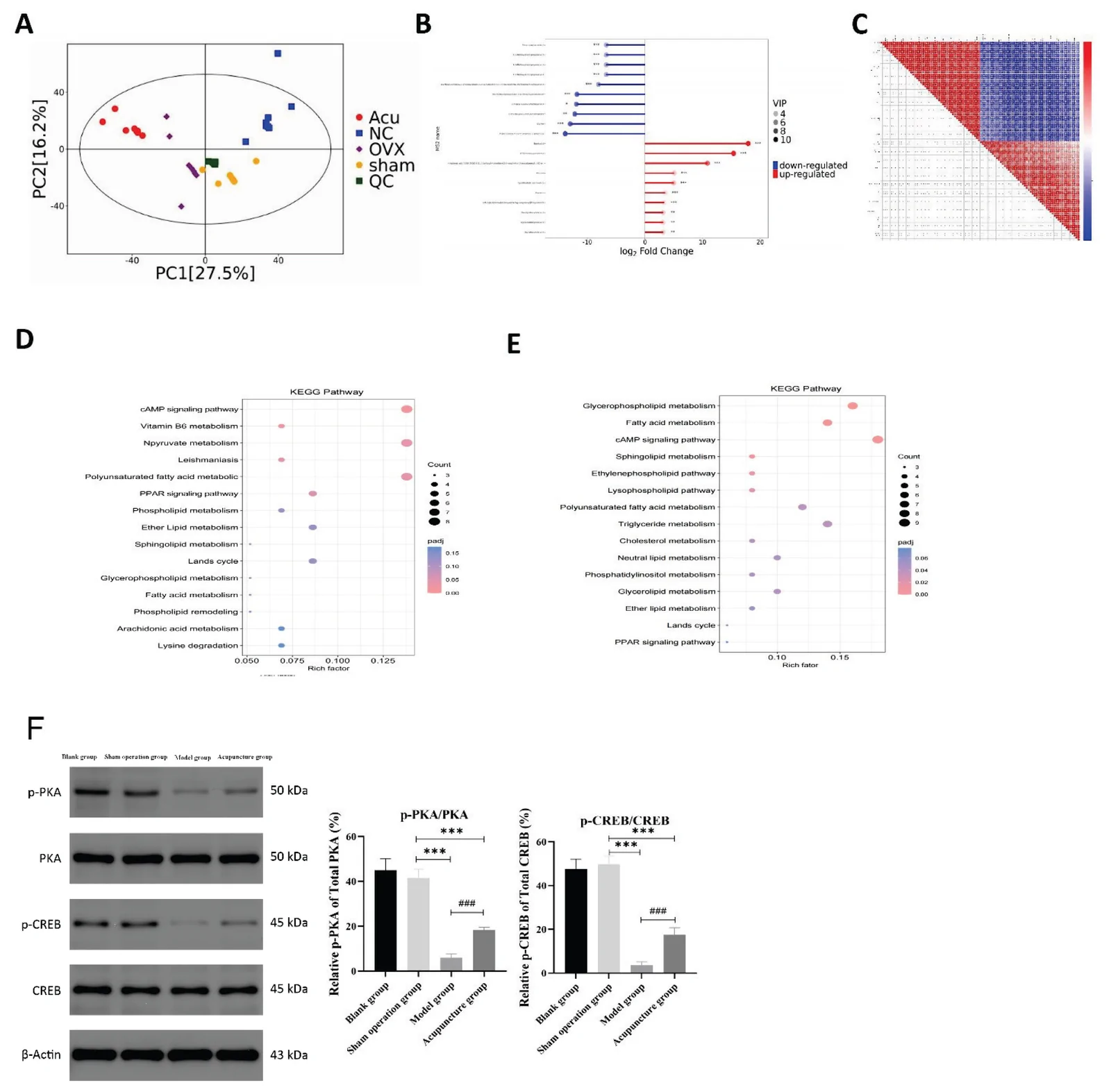
Effects of acupuncture on the cAMP-PKA-CREB pathway. (**A–B**) PCA and PLS-DA score plots. (**C**) Heat map of differential metabolites. (**D**) KEGG pathway enrichment analysis of differential metabolites between the Blank and Model groups. The x-axis represents the Rich Factor (ratio of differential metabolites to total metabolites in the pathway), and the y-axis represents the pathway name. The size of the dots is positively correlated with the number of differential metabolites, and the color represents the P-value (redder color indicates more significant enrichment). (**E**) KEGG pathway enrichment analysis of differential metabolites between the Model and Acupuncture treatment groups. (**F**) Western blot and quantification of p-PKA/PKA and p-CREB/CREB. Data are mean ± SD, n=8. *** P<0.001.

Metabolomics analysis revealed distinct metabolic profiles among groups. The PCA (Fig. 6A) and PLS-DA (Fig. 6B) score plots showed clear separation, indicating significant metabolic alterations in PMOP rats and the regulatory effect of acupuncture. A total of 50 differential metabolites were identified and visualized in a heat map (Fig. 6C). KEGG pathway enrichment analysis further elucidated the potential mechanisms. Both the Blank vs. Model (Fig. 6D) and Model vs. Acupuncture (Fig. 6E) comparisons showed that differential metabolites were significantly enriched in lipid metabolism pathways (e.g., glycerophospholipid, fatty acid metabolism) and the cAMP signaling pathway. Integrated analysis indicates that acupuncture improves osteoporosis likely by modulating these metabolic networks. Consistently, Western blot results confirmed that acupuncture upregulated the phosphorylation of PKA and CREB (Fig. 6F), validating the activation of the cAMP-PKA-CREB pathway.

## Discussion

This comprehensive study investigated the multi-faceted effects and mechanisms of acupuncture on bone and lipid metabolism in a PMOP rat model. The results demonstrate that acupuncture effectively improves bone density (after adjusting for femoral shaft thickness) and bone tissue microarchitecture with significant improvements in quantitative histomorphometric para-meters, regulates bone metabolism balance, simultaneously improves lipid metabolism disorders and inflammatory status, partially restores systemic estrogen, and optimizes gut microbiota composition. These beneficial effects appear to be mediated, at least in part, through activation of the cAMP-PKA-CREB signaling pathway.

The successful establishment of the PMOP rat model was confirmed by significantly decreased femoral BMD, deteriorated trabecular bone structure with quantitatively measured reductions in Tb.N, Tb.Th, and BV/TV, reduced serum E2 levels, and increased bone turnover markers. An important methodological refinement in this study was the adjustment of BMD values for femoral shaft thickness, addressing the limitation that dual-energy X-ray absorptiometry measures areal rather than volumetric bone density. This adjustment revealed that acupuncture’s beneficial effects on BMD were independent of changes in bone size, strengthening the validity of our findings.

The quantitative histomorphometric analysis provides objective evidence for acupuncture’s protective effects on trabecular bone microarchitecture. The significant improvements in Tb.N, Tb.Th, and BV/TV in the acupuncture group indicate that acupuncture not only prevents bone loss but may also promote bone formation. These findings align with our bone turnover marker results, where acupuncture increased OPG and decreased RANKL, β-CTX, and PINP levels, suggesting a shift toward bone anabolism. The OPG/RANKL system is a key regulator of bone remodeling, with increased OPG/RANKL ratio favoring bone formation over resorption [25,26].

Acupuncture has been shown to elevate estrogen levels – particularly estradiol – as well as progesterone, prolactin, and other related hormones [27]. A particularly intriguing finding of the present study was the restoration of serum E2 levels in the acupuncture group despite bilateral ovariectomy. The serial E2 measurements demonstrated a progressive increase from week 4 onwards, suggesting that acupuncture may stimulate alternative sources of estrogen production, such as adrenal glands or adipose tissue. Importantly, while acupuncture partially restored E2 levels, they remained significantly lower than the sham group, indicating that the bone-protective effects likely involve multiple mechanisms beyond estrogen replacement alone.

Regarding the important question of whether acupuncture combined with estrogen suppression therapy (such as in combined treatment scenarios) might counteract the beneficial effects, we must acknowledge that our current study design does not directly address this scenario. In the ovariectomy model, we observed that acupuncture can partially restore E2 levels through potential extragonadal mechanisms. However, whether these benefits would persist when pharmacological estrogen inhibition is simultaneously imposed remains to be investigated. Future studies should be designed to include an acupuncture control group as well as a combined acupuncture plus estrogen inhibition group, in order to determine whether the bone-protective effects of acupuncture are sufficiently independent of the estrogen pathway. This has important clinical implications for patients who require long-term estrogen suppression. This has important clinical implications for patients who require long-term estrogen suppression.

The strong negative correlation between BMI reduction and BMD improvement (r=−0.68, P=0.003) in the acupuncture group suggests that metabolic modulation plays a significant role in acupuncture’s bone-protective effects. This finding aligns with the concept that lipid metabolism and bone metabolism are closely interconnected through shared signaling pathways and endocrine factors [15,16]. The correlation analysis indicates that acupuncture’s effects on reducing adiposity may contribute to improved bone health, potentially through reduced inflammatory burden and altered adipokine secretion.

The gut microbiota analysis revealed a novel mechanism through which acupuncture may exert its bone-protective effects. The restoration of beneficial bacteria (Lactobacillus, Bifidobacterium, Akkermansia) and reduction of pathogenic species represents a significant finding, as emerging evidence suggests that gut microbiota plays a crucial role in bone metabolism through the gut-bone axis. Lactobacillus and Bifidobacterium species have been shown to produce short-chain fatty acids and regulate immune responses, which can influence bone remodeling. The strong positive correlation between Lactobacillus abundance and BMD (r=0.72, P<0.001), as well as negative correlations with inflammatory markers, suggests that gut microbiota modulation may be an important mechanism underlying acupuncture’s anti-osteoporotic effects. Furthermore, the positive correlation between Bifidobacterium abundance and E2 levels (r=0.58, P<0.01) raises the intriguing possibility that gut microbiota may be involved in modulating estrogen metabolism or extragonadal estrogen production, potentially contributing to the E2 restoration observed in the acupuncture group.

The activation of the cAMP-PKA-CREB pathway in the acupuncture group provides molecular insight into the mechanisms of action. This pathway is critical for both osteoblast differentiation and adipocyte regulation [19–22]. The upregulation of phosphorylated PKA and CREB suggests that acupuncture may enhance osteogenic differentiation while potentially inhibiting adipogenic differentiation, consistent with the observed improvements in both bone parameters and lipid metabolism. The integration of metabolomics data further supports the involvement of this pathway, with differential metabolites primarily involving lipid metabolism pathways that are known to interact with cAMP signaling.

Taken together, these findings suggest that acupuncture exerts its therapeutic effects on PMOP through multiple interconnected mechanisms: (1) partial restoration of estrogen levels through potential extragonadal mechanisms, (2) regulation of bone turnover markers favoring bone formation, (3) modulation of systemic metabolism reducing adiposity and improving lipid profiles, (4) attenuation of chronic inflammation, (5) optimization of gut microbiota composition and function, and (6) activation of the cAMP-PKA-CREB signaling pathway. The interplay among these mechanisms likely contributes to the comprehensive therapeutic effects observed.

Several limitations of this study should be acknowledged. First, the exact mechanism by which acupuncture restored E2 levels and promoted ovarian follicular development after complete ovariectomy remains unclear and requires further investigation, including detailed examination for potential residual ovarian tissue and assessment of extragonadal estrogen synthesis capacity. Second, while we observed gut microbiota changes associated with acupuncture treatment, causality has not been established. Future studies using fecal microbiota transplantation or germ-free animal models would help determine whether gut microbiota modulation is a primary mechanism or a secondary consequence of acupuncture’s effects. Third, the clinical translation of our findings requires validation, as rat models may not fully recapitulate human PMOP pathophysiology. Fourth, longer-term follow-up studies are needed to assess the durability of acupuncture’s effects and determine optimal treatment protocols. Finally, as discussed above, future studies should investigate whether acupuncture’s beneficial effects are maintained when combined with pharmacological estrogen suppression, which would be clinically relevant for patients requiring such treatment.

Despite these limitations, our study provides comprehensive evidence that acupuncture is an effective intervention for PMOP through multiple mechanisms involving hormonal regulation, metabolic modulation, inflammatory control, gut microbiota optimization, and cAMP-PKA-CREB pathway activation. These findings support acupuncture as a promising complementary or alternative therapy for PMOP and provide a scientific foundation for its clinical application. Future research should focus on elucidating the detailed molecular mechanisms, particularly regarding ovarian tissue regeneration and gut-bone axis modulation, and conducting well-designed clinical trials to validate these preclinical findings in human PMOP patients.

## Supplementary Information







## References

[1] Walker MD, Shane E (2023). Postmenopausal Osteoporosis. N Engl J Med.

[2] Subarajan P, Arceo-Mendoza RM, Camacho PM (2024). Postmenopausal Osteoporosis: A Review of Latest Guidelines. Endocrinol Metab Clin North Am.

[3] Muñoz M, Robinson K, Shibli-Rahhal A (2020). Bone Health and Osteoporosis Prevention and Treatment. Clin Obstet Gynecol.

[4] Gao SS, Zhao YF (2023). Quality of life in postmenopausal women with osteoporosis: a systematic review and meta-analysis. Qual Life Res.

[5] Kubi JA, Brah AS, Cheung KMC, Chen ACH, Lee YL, Lee K-F (2024). Low-molecular-weight estrogenic phytoprotein suppresses osteoporosis development through positive modulation of skeletal estrogen receptors. Bioact Mater.

[6] Fischer V, Haffner-Luntzer M (2022). Interaction between bone and immune cells: Implications for postmenopausal osteoporosis. Semin Cell Dev Biol.

[7] Yang YX, Zhang YW, Kuang SS, Zhang Y, Zhou LD, Mao B-Y, Xie J-S (2024). Studies on the Anti-Inflammatory Effect of Xiaoyao Pills in The Treatment of Postmenopausal Osteoporosis in Mice. J Vis Exp.

[8] Yao Y, Cai X, Chen Y, Zhang M, Zheng C (2025). Estrogen deficiency-mediated osteoimmunity in postmenopausal osteoporosis. Med Res Rev.

[9] Brown JP (2021). Long-Term Treatment of Postmenopausal Osteoporosis. Endocrinol Metab (Seoul).

[10] ACOG Committee on Clinical Practice Guidelines-Gynecology (2022). Management of Postmenopausal Osteoporosis: ACOG Clinical Practice Guideline No. 2. Obstet Gynecol.

[11] Kanis JA, McCloskey EV, Johansson H, Reginster JY, Scientific Advisory Board of the European Society for Clinical and Economic Aspects of Osteoporosis (ESCEO) and the Committees of Scientific Advisors and National Societies of the International Osteoporosis Foundation (IOF) (2019). European guidance for the diagnosis and management of osteoporosis in postmenopausal women. Osteoporos Int.

[12] Kim YJ (2024). How You Treat Groin Pain in the Adult Patient with Acupuncture and/or Chinese Herbs. Med Acupunct.

[13] Ge J, Wang H, Zheng H, Luo Y, Wang J (2020). Expert consensus on Chinese medicine prevention and treatment of primary osteoporosis. Chin J Osteopor.

[14] Fan H, Ji F, Lin Y, Zhang M, Qin W, Zhou Q, Wu Q (2016). Electroacupuncture stimulation at CV4 prevents ovariectomy-induced osteoporosis in rats via Wnt/β-catenin signaling. Mol Med Rep.

[15] Zhang LG, Liu QL, Zeng XS, Gao W, Niu Y, MX (2021). Association of dyslipidaemia with osteoporosis in postmenopausal women. J Int Med Res.

[16] Patsch JM, Li X, Baum T, Yap SP, Karampinos DC, Schwartz AV, Link TM (2013). Bone marrow fat composition as a novel imaging biomarker in postmenopausal women with prevalent fragility fractures. J Bone Miner Res.

[17] Kawai M, de Paula FJ, Rosen CJ (2012). New insights into osteoporosis: the bone-fat connection. J Intern Med.

[18] Mundy GR (2007). Osteoporosis and inflammation. Nutr Rev.

[19] Marie PJ (2012). Signaling pathways affecting skeletal health. Curr Osteoporos Rep.

[20] Rosen ED, MacDougald OA (2006). Adipocyte differentiation from the inside out. Nat Rev Mol Cell Biol.

[21] Zhang R, Edwards JR, Ko SY, Dong S, Liu H, Oyajobi Bo (2011). Transcriptional regulation of BMP2 expression by the PTH-CREB signaling pathway in osteoblasts. PLoS One.

[22] Fox KE, Fankell DM, Erickson PF, Majka SM, Crossno JT, Klemm DJ (20026). Depletion of cAMP-response element-binding protein/ATF1 inhibits adipogenic conversion of 3T3-L1 cells ectopically expressing CCAAT/enhancer-binding protein (C/EBP) alpha, C/EBP beta, or PPAR gamma 2. J Biol Chem.

[23] Yong T, Yan L, Miao Z, Yanjun D, Xiaofang Y (2021). Experimental Acupuncture Science.

[24] Bouxsein ML, Boyd SK, Christiansen BA, Guldberg RE, Jepsen KJ, Müller R (2010). Guidelines for assessment of bone microstructure in rodents using micro-computed tomography. J Bone Miner Res.

[25] Boyce BF, Xing L (2008). Functions of RANKL/RANK/OPG in bone modeling and remodeling. Arch Biochem Biophys.

[26] Hofbauer LC, Schoppet M (2004). Clinical implications of the osteoprotegerin/RANKL/RANK system for bone and vascular diseases. JAMA.

[27] Ko JH, Kim SN (2018). A Literature Review of Women’s Sex Hormone Changes by Acupuncture Treatment: Analysis of Human and Animal Studies. Evid Based Complement Alternat Med.

