# Supplementary material for: Effects and Mechanisms of Action of Acupuncture on Bone and Lipid Metabolism in a Rat Model of Postmenopausal Osteoporosis
Source: Physiol Res. 2026 Jun 1;75(3):507–19. doi: 10.33549/physiolres.935700 (PMC13557543; doi:10.33549/physiolres.935700)
Supplement: Supplementary file 1 [file 75_507_Suppl_Table_1.docx]

**Supplementary Table 1.** Bone mineral density, femoral shaft thickness, and serum bone metabolism markers (x̄ ± SD).

| *Parameter* | **Blank Group** | **Sham Operation Group** | **Model Group** | **Acupuncture Group** | **F value** | **P value** |
| --- | --- | --- | --- | --- | --- | --- |
| *Baseline measurements* |  |  |  |  |  |  |
| *BMD (g/cm^2^)* | 0.203±0.007 | 0.204±0.006 | 0.202±0.008 | 0.203±0.007 | 0.42 | 0.74 |
| *Femoral shaft thickness (mm)* | 1.85±0.12 | 1.87±0.11 | 1.86±0.13 | 1.84±0.12 | 0.18 | 0.91 |
| *3-month measurements* |  |  |  |  |  |  |
| *Raw BMD (g/cm^2^)* | 0.206±0.008 | 0.204±0.006 | 0.153±0.015*** | 0.179±0.010***^###^** | 233.7 | <0.001 |
| *Femoral shaft thickness (mm)* | 1.88±0.09 | 1.88±0.10 | 1.62±0.14** | 1.74±0.13***^#^** | 28.4 | <0.001 |
| *Adjusted BMD (g/cm^2^)* | 0.206±0.008 | 0.204±0.006 | 0.156±0.014*** | 0.184±0.011***^##^** | 185.6 | <0.001 |
| *OPG (pg/ml)* | 1058.06±278.60 | 1057.90±261.82 | 229.42±107.68*** | 691.90±213.06**^###^** | 24.348 | <0.001 |
| *RANKL (pg/ml)* | 61.89±22.07 | 64.57±22.41 | 187.82±39.37*** | 109.80±31.59**^##^** | 31.279 | <0.001 |
| *β-CTX (ng/ml)* | 6.81±2.00 | 6.90±1.87 | 16.10±3.97*** | 12.17±3.04**^##^** | 20.811 | <0.001 |
| *PINP (ng/ml)* | 20.46±6.78 | 19.31±7.73 | 67.87±15.94*** | 44.39±9.36**^##^** | 37.898 | <0.001 |

Data are presented as mean ± SD, n=8. *** P<0.001, ** P<0.01, * P<0.05 vs. Sham operation group; **^###^** P<0.001, **^##^** P<0.01, **^#^** P<0.05 vs. Model group.
