# Supplementary material for: Effects and Mechanisms of Action of Acupuncture on Bone and Lipid Metabolism in a Rat Model of Postmenopausal Osteoporosis
Source: Physiol Res. 2026 Jun 1;75(3):507–19. doi: 10.33549/physiolres.935700 (PMC13557543; doi:10.33549/physiolres.935700)
Supplement: Supplementary file 2 [file 75_507_Suppl_Table_2.docx]

**Supplementary Table 2.** Statistical analysis of BMI in each group of rats at different time points (x̄ ± SD, g/cm^2^).

|  | **Adaptation** | **Modeling** | **Treatment** | | | | | | | | | | | | |
| --- | --- | --- | --- | --- | --- | --- | --- | --- | --- | --- | --- | --- | --- | --- | --- |
| *Week* | 1 | 2 | 3 | 4 | 5 | 6 | 7 | 8 | 9 | 10 | 11 | 12 | 13 | 14 | 15 |
| ***A:*** *Blank group* | 0.783± 0.01 | 0.767± 0.015 | 0.76±0.017 | 0.748±0.018 | 0.737±0.017 | 0.726±0.014 | 0.721±0.015 | 0.713±0.009 | 0.696±0.011 | 0.693±0.016 | 0.681±0.014 | 0.669±0.011 | 0.662±0.011 | 0.654±0.016 | 0.655±0.011 |
| ***B:*** *Sham ope-ration group* | 0.773± 0.012 | 0.771± 0.012 | 0.766±0.01 | 0.757±0.016 | 0.756±0.019 | 0.745±0.014 | 0.722±0.012 | 0.703±0.011 | 0.686±0.013 | 0.683±0.013 | 0.666±0.013 | 0.661±0.008 | 0.654±0.011 | 0.651±0.012 | 0.658±0.013 |
| ***C:*** *Model group* | 0.761± 0.016 | 0.776± 0.013 | 0.836±0.014 | 0.884±0.03 | 0.925±0.015 | 0.952±0.015 | 0.977±0.019 | 0.992±0.021 | 1.015±0.025 | 1.03±0.025 | 1.061±0.033 | 1.084±0.028 | 1.111±0.028 | 1.123±0.026 | 1.148±0.022 |
| ***D:*** *Acupuncture group* | 0.759± 0.012 | 0.769± 0.011 | 0.831±0.012 | 0.885±0.01 | 0.915±0.013 | 0.919±0.023 | 0.909±0.041 | 0.906±0.041 | 0.907±0.037 | 0.903±0.038 | 0.895±0.034 | 0.885±0.024 | 0.881±0.027 | 0.863±0.026 | 0.854±0.02 |
| *P value B vs. C* | 0.091 | 0.107 | <0.001 | <0.001 | <0.001 | <0.001 | <0.001 | <0.001 | <0.001 | <0.001 | <0.001 | <0.001 | <0.001 | <0.001 | <0.001 |
| *P value B vs. D* | 0.025 | 0.419 | <0.001 | <0.001 | <0.001 | <0.001 | <0.001 | <0.001 | <0.001 | <0.001 | <0.001 | <0.001 | <0.001 | <0.001 | <0.001 |
| *P value C vs. D* | 0.347 | 0.115 | 0.067 | 0.447 | 0.076 | 0.001 | <0.001 | <0.001 | <0.001 | <0.001 | <0.001 | <0.001 | <0.001 | <0.001 | <0.001 |
