# Supplementary material for: Effects and Mechanisms of Action of Acupuncture on Bone and Lipid Metabolism in a Rat Model of Postmenopausal Osteoporosis
Source: Physiol Res. 2026 Jun 1;75(3):507–19. doi: 10.33549/physiolres.935700 (PMC13557543; doi:10.33549/physiolres.935700)
Supplement: Supplementary file 3 [file 75_507_Suppl_Table_3.docx]

**Supplementary Table 3.** Statistical analysis of blood lipid levels and inflammatory factors in each group of rats (x̄ ± SD).

|  | **TC**  (mmol/l) | **TG**  (mmol/l) | **LDL-C**  (mmol/l) | **HDL-C**  (mmol/l) | **E2**  (pmol/l) | **IL-6**  (pg/ml) | **TNF-α**  (pg/ml) |
| --- | --- | --- | --- | --- | --- | --- | --- |
| *Blank group* | 2.36±0.40 | 0.93±0.25 | 1.04±0.28 | 1.89±0.47 | 200.28±33.22 | 31.58±11.94 | 52.99±18.99 |
| *Sham operation group* | 2.32±0.41 | 0.90±0.33 | 0.99±0.29 | 1.87±0.50 | 197.36±31.82 | 34.13±13.12 | 47.91±19.48 |
| *Model group* | 4.68±0.78 | 2.11±0.14 | 2.13±0.46 | 0.87±0.36 | 52.50±27.09 | 124.20±27.14 | 154.85±34.28 |
| *Acupuncture group* | 3.27±0.38 | 1.48±0.26 | 1.45±0.24 | 1.33±0.42 | 110.35±37.47 | 75.69±26.20 | 113.54±27.80 |
| *F value* | 35.779 | 40.065 | 20.359 | 9.765 | 38.761 | 34.863 | 31.257 |
| *P value* | <0.001 | <0.001 | <0.001 | <0.001 | <0.001 | <0.001 | <0.001 |
